# Novel Tricyclic Flavonoids as Promising Anti-MRSA Agents

**DOI:** 10.3390/ph17101276

**Published:** 2024-09-26

**Authors:** Cristina-Veronica Moldovan, Loredana-Elena Mantea, Mihaela Savu, Peter G. Jones, Laura Gabriela Sarbu, Marius Stefan, Mihail Lucian Birsa

**Affiliations:** 1 Department of Biology, Faculty of Biology, Alexandru Ioan Cuza University of Iasi, Bd. Carol I, No. 11, 700506 Iasi, Romania; mcristina.veronica@gmail.com (C.-V.M.); mantealoredana9@gmail.com (L.-E.M.); mihaelasavu2@gmail.com (M.S.); 2 Institute of Inorganic and Analytical Chemistry, Technical University of Braunschweig, Hagenring 30, D-38106 Braunschweig, Germany; p.jones@tu-braunschweig.de; 3 Department of Chemistry, Alexandru Ioan Cuza University of Iasi, No. 11 Carol I Blvd., 700506 Iasi, Romania; laura.sarbu@uaic.ro

**Keywords:** synthetic flavonoids, benzopyran, new anti-MRSA agents, bacteriostatic, bactericidal, antimicrobial resistance

## Abstract

**Background:** Methicillin-resistant *Staphylococcus aureus* (MRSA) is considered the main cause of nosocomial and community-associated infections. Because of antimicrobial resistance, MRSA infections are difficult or impossible to treat, leading to high mortality rates and significant economic and societal costs. In view of the MRSA challenge to public health all over the world, the identification of new and effective anti-MRSA agents is a high medical priority. **Objectives:** A new series of tricyclic flavonoids with a methyl substituent on ring A of the flavonoid skeleton was synthesized to assess their antimicrobial properties. **Methods:** The structures of novel synthetic tricyclic flavonoids and their 3-dithiocarbamic flavanones were proven by X-ray structural analyses. Minimum inhibitory concentration (MIC) and minimum bactericidal/fungicidal concentration (MBC/MFC) were used to evaluate antimicrobial activity. Growth kinetic and time–kill assays were employed to confirm the antibacterial effectiveness. The mechanism of action was investigated using fluorescence microscopy. **Results:** Our results show that the tricyclic flavonoids exhibited important antibacterial and antifungal activities, with MIC and MBC values as low as 1.95 µg/mL and 3.90 µg/mL recorded for compound **5e** against a multidrug-resistant MRSA strain. Flavonoid **5e** induced a more important bacteriostatic effect compared with chloramphenicol, inhibiting the bacterial growth for up to 24 h at concentrations equivalent to 2 × MIC. Also, **5e** exhibited a significant bactericidal activity, with no viable cells evidenced after 6 h of incubation in the presence of MBC and a total kill effect recorded up to 24 h. The anti-MRSA activity may be explained by the cell membrane impairment induced by **5e**. **Conclusions:** All the data support the idea that flavonoid **5e** is a reliable candidate to develop effective anti-MRSA agents, but further studies are necessary.

## 1. Introduction

Methicillin-resistant *Staphylococcus aureus* (MRSA) is a Gram-positive bacterium resistant to different antibiotics, including methicillin and other beta-lactams. MRSA is considered as the main cause of nosocomial infections. However, recent data have shown that it is also an important source of community-associated infections [1]. MRSA is responsible for many types of infections, including skin, lung, heart and bloodstream infections [2]. Because of antimicrobial resistance (AMR), MRSA infections are more difficult or impossible to treat, leading frequently to sepsis and death, especially in hospitalized patients. As a result, high morbidity and mortality rates and significant economic and societal costs are recorded [3]. According to Murray et al., in 2019, MRSA was responsible for more than 100,000 deaths attributable to AMR [4]. Furthermore, the alarming spread of MRSA on all continents during the last three decades is a major medical concern [5]. Therefore, MRSA is considered a significant challenge to public health all over the world [6].

MRSA exhibits AMR through several mechanisms including production of penicillin-binding protein 2a (PBP2a), alteration of other PBPs, beta-lactamase production, efflux pumps, regulation of resistance genes, or acquisition of new resistance genes [7]. The continuous emergence of new antibiotic-resistant MRSA strains poses serious clinical challenges, threatening the effectiveness of treatment options. As a consequence, many of the antibiotics currently used in therapy, including vancomycin, considered to be the mainstay of severe MRSA infection treatment [8], are becoming less and less efficient. Moreover, MRSA resistance extends to newer antibiotics such as linezolid and daptomycin, which are no longer seen as effective solutions [9].

Considering the significant MRSA global burden, the World Health Organization (WHO) has identified MRSA as a high-priority pathogen, emphasizing the urgent need for the development of new antibacterial agents that can effectively target MRSA without promoting further resistance [10]. An alternative approach to identify new anti-MRSA agents could involve compounds with known antibacterial activity, such as flavonoids. Flavonoids are naturally occurring compounds found in various plant products well known for their health benefits [11]. The great diversity of chemical properties provides them with the ability to combat a wide range of pathogens such as bacteria, fungi, and viruses [12]. On the other hand, synthetic flavonoids have gained significant attention for their enhanced antimicrobial potency, improved stability and bioavailability, and overcoming resistance, making them ideal candidates for the development of new efficient antimicrobial agents against multidrug-resistant microorganisms [13].

Our research group has focused in recent years on a new class of sulfur-containing tricyclic flavonoids with halogen substituents at the benzopyran core. We previously showed that those compounds displayed important antibacterial activity against Gram-positive and negative strains, including ESKAPE pathogens [14,15]. Here we report the synthesis and characterization of new synthetic tricyclic flavonoids with a lipophilic substituent (a methyl group) on ring A. Theoretically, adding a new methyl group should enhance the activity against Gram-negative bacteria [16]; we therefore intended to perform a structure relationship study using different microbial strains. Also, we assessed the effectiveness of these flavonoids against a multidrug-resistant clinical MRSA isolate with the aim of identifying new antibacterial compounds that could mitigate the burden of the alarming emergence of antibiotic-resistant MRSA strains.

## 2. Results and Discussion

### 2.1. Synthesis

**Scheme 1:**
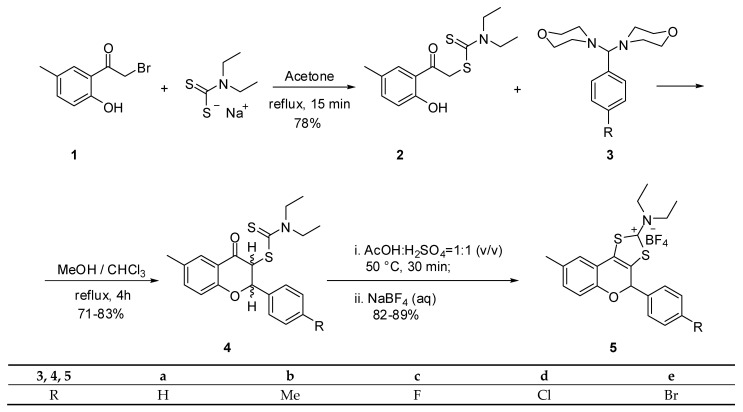
The synthesis of tricyclic flavonoids **5a–e**.

Substituted 4-chromanones and flavanones have been reported to be obtained by the reaction of dithiocarbamates of type **2** with aminals **3** [17]. Based on our previous findings, we decided to extend our studies to a new class of tricyclic flavonoids with a structure related to the compounds depicted in Scheme 1. Thus, 3-dithiocarbamic flavanones **4** were synthesized by reacting 1-(2-hydroxy-5-methylphenyl)-1-oxaethan-2-yl *N*,*N*-diethylaminocarbodithioate (**2**) with aminals **3** (Scheme 1). The latter compounds were obtained by reacting the corresponding substituted benzaldehydes with morpholine. The aminals are more stable and reactive compounds than free aldehydes. In principle, the reaction mechanism involved the formation of a carbon–carbon bond by elimination of morpholine from aminal and a hydrogen atom from the α position toward the carbonyl group of phenacyl dithiocarbamates **2**. The released morpholine acts as a base-generating phenolate anion that undergoes a nucleophilic attack replacing the second morpholine unit from the original aminal and closes the benzopyran ring.

**Figure 1:**
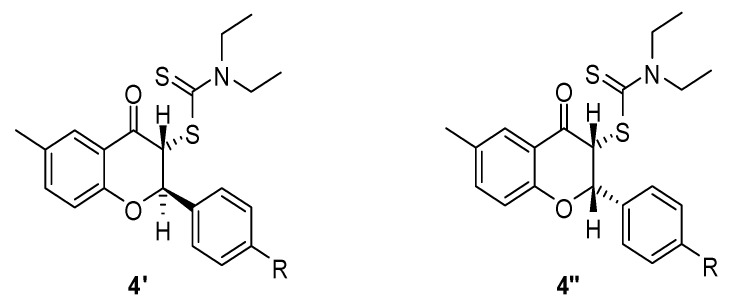
Diastereoisomers of flavonones **4.**

The original short communication on the synthesis of flavanones of type **4** [17] did not mention the existence of diastereoisomers. However, we identified an inseparable mixture of two diastereoisomers. In principle, these flavanones should have two diastereoisomers depending on the relative orientation of the H-2 and H-3 hydrogen atoms of the flavanones **4**. These two hydrogen atoms can point either to opposite sides or to the same side of the C-2 and C-3 carbon atoms in the benzopyran ring. Based on the magnitude of their coupling constants, the two diastereoisomers were easily identified. Moreover, it is reasonable to assume that the most stable isomer is that with an *anti* orientation of the two hydrogen atoms **4′** rather than the *syn* diastereomer **4″** (Figure 1). Our assumption is based on the well-known higher stability of less sterically hindered isomers. This means that, as in the case of *cis* and *trans* isomers, the most thermodynamically stable one is the less hindered *anti* diastereomer. The diastereoisomeric ratio and coupling constants of flavonones **4** are presented in Table 1. For all flavanones, the major isomer is the *anti* one, as it is the most thermodinamically stable.

**Figure 2:**
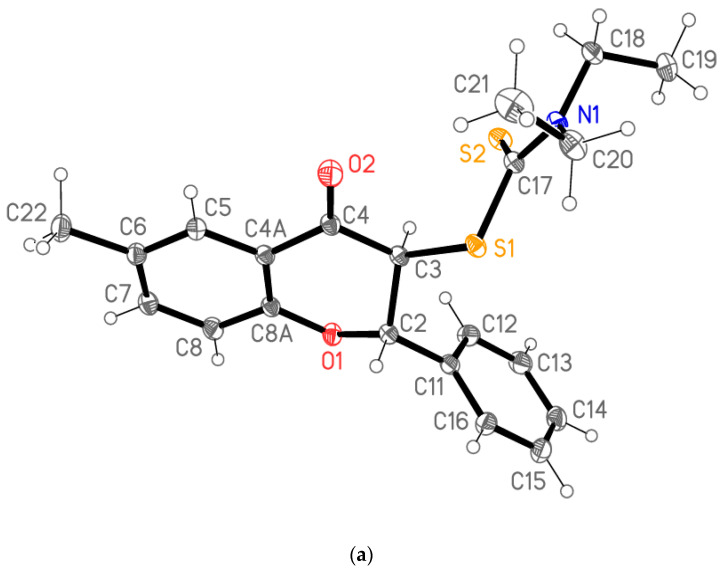

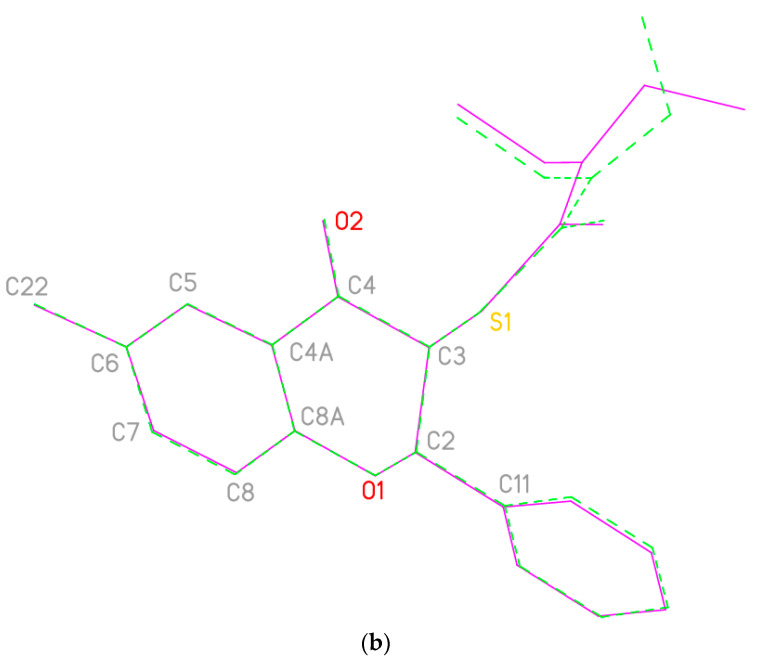
(**a**). Structure of one of the two independent molecules of flavanone **4a**; ellipsoids represent 50% probability levels. (**b**). Least-squares fit of the two independent molecules of flavanone **4a**. Hydrogen atoms are omitted. Fitted atoms (r.m.s. deviation 0.03 Å) are labeled.

The structural information provided by NMR data was unambiguously confirmed by X-ray analysis (see Supplementary Materials). The structure of the *anti* diastereomer of flavanone **4a** is presented in Figure 2a. The asymmetric unit consists of two molecules that differ in the orientation of the diethylaminocarbodithioate groups (Figure 2b).

**Figure 3:**
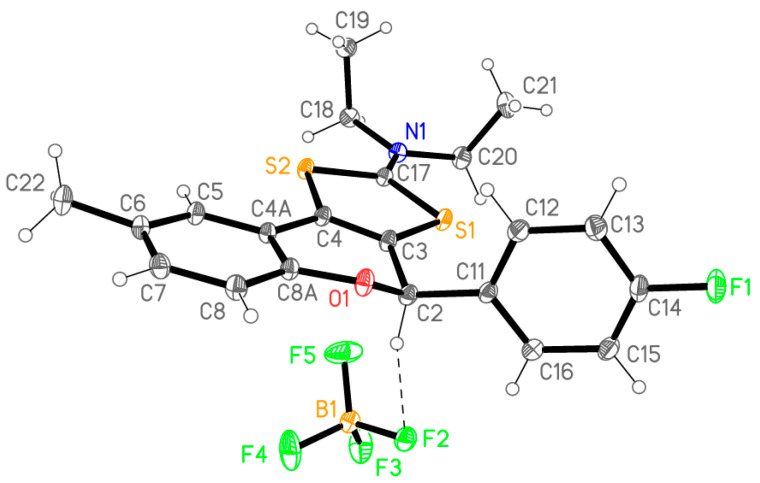
Molecular structure of flavanoid **5c**; ellipsoids represent 50% probability levels. The dashed line shows a short hydrogen bond H2⋯F2 of 2.36 Å between cation and anion; a further short contact H5⋯F5 of 2.30 Å connects the ions to form inversion-symmetric dimers.

The most accessible approach for the synthesis of 1,3-dithiolium-2-yl cations involves the heterocyclization of the corresponding phenacyl dithiocarmates under acid catalysis. The cyclization of flavanones **4** should hence provide the new tricyclic flavonoids **5** using the conditions described in Scheme 1. Thus, using a concentrated sulfuric acid–glacial acetic acid (1:1 *v*/*v*) mixture, at 50 °C, the cyclization of dithiocarbamates **4** takes place under mild reaction conditions. The acid cyclization of dithiocarbamic flavanones **4** involves in a first step the activation of the carbonyl group toward nucleophiles by protonation of the oxygen atom. The heterocyclization takes place by nucleophilic attack of the sulfur atom to the protonated carbonyl group, followed by water elimination that leads to the formation of a fused 1,3-dithiolium ring. The addition of an aqueous solution of sodium tetrafluoroborate provides tricyclic flavonoids **5** as white crystalline compounds in good yields (83–89%). The structures of flavonoids **5** were confirmed by analytical and spectral data (see Supplementary Materials). Moreover, these data were confirmed by X-ray analysis as shown in Figure 3 for the structure of flavonoid **5c**.

### 2.2. In Vitro Assessment of Antimicrobial Susceptibility

The tested flavonoids were significantly more active against Gram-positive bacteria than against Gram-negative strains, as shown in Table 2. The MIC values recorded against Gram-positive strains ranged from 1.95 to 62.50 µg/mL, with flavonoids **5e**, **5d,** and **5b** as the most active compounds. The highest antibacterial activity was recorded for compound **5e** against *S. aureus* medbio1-2012 (MIC = 1.95 µg/mL). The Gram-negative strains were not susceptible to compounds **5a**–**e**, the recorded activity being comparable with the negative control. One exception occurred regarding compound **5e**, for which an MIC value of 62.50 µg/mL was registered against *E. coli*. Regarding the antifungal activity, only flavonoids **5b**, **5d,** and **5e** showed inhibitory effects on the growth of the two tested *Candida* strains (MIC = 31.25 µg/mL).

The synthetic flavonoids also showed important bactericidal activity against Gram-positive bacteria, with one strain (*S. aureus* prxbio2) considered as less sensitive. The lowest MBC values of 3.90 and 7.81 µg/mL were recorded for compounds **5e** and **5b** against an MRSA strain (*S. aureus* medbio1-2012)—Table 3. No significant bactericidal activity was registered against Gram-negative tested strains. Among the five synthetic flavonoids, compounds **5b** and **5e** showed the highest fungicidal activity against a *C. krusei* strain resistant to fluconazol (MFC = 31.25 µg/mL).

Based on MIC and MBC values, flavonoid **5e** was selected for further tests to assess the antibacterial effectiveness against the most susceptible test microorganism—a clinical multidrug-resistant MRSA isolate (*S. aureus* medbio1-2012).

### 2.3. Flavonoid ***5e*** Induced Important Bacteriostatic Effect against MRSA

**Figure 4:**
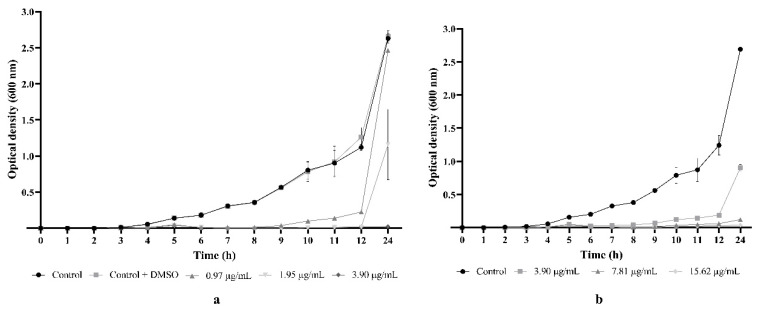
Dynamics of *Staphylococcus aureus* medbio1-2012 growth in the presence of **5e** (**a**) and chloramphenicol (**b**). MIC of **5e** = 1.95 µg/mL; MIC of chloramphenicol = 7.81 µg/mL. MRSA cells incubated in MHB served as growth control, and MHB supplemented with DMSO served as negative control. Values are expressed as means of three independent experiments. Bars indicate SEM.

The effect of **5e** and chloramphenicol on *S. aureus* medbio1-2012 cells during the exponential phase of growth is presented in Figure 4a,b. No significant differences were registered during the 24 h incubation time between the growth of MRSA cells incubated in MHB (growth control) and MHB supplemented with DMSO (negative control). Different concentrations of **5e** induced significant bacteriostatic effects over time against the MRSA-tested strain compared to controls. Thus, incubation of the cells in the MHB medium supplemented with **5e** at ½ MIC resulted in a 5 h growth delay and a significantly reduced growth up to 12 h compared to the controls. A total inhibition of MRSA growth was recorded at a concentration equivalent to an MIC up to 12 h. However, after 24 h of incubation, the spectrophotometric measurements revealed bacterial growth in the presence of both **5e** and chloramphenicol. Furthermore, no MRSA growth was evidenced at a concentration equivalent to 2 × MIC during the entire span of the experiment, as the growth curve depicted in Figure 4a shows.

The growth curve analysis revealed that increasing concentrations of **5e** resulted in a more pronounced bacteriostatic effect, suggesting that the MRSA growth was inhibited by the tested flavonoid in a dose- and time-dependent manner. Moreover, the bacteriostatic effects recorded for **5e** and chloramphenicol (an antibiotic to which the MRSA strain was sensitive) were similar for all tested concentrations.

### 2.4. Flavonoid ***5e*** Is a Potent Bactericidal Compound

**Figure 5:**
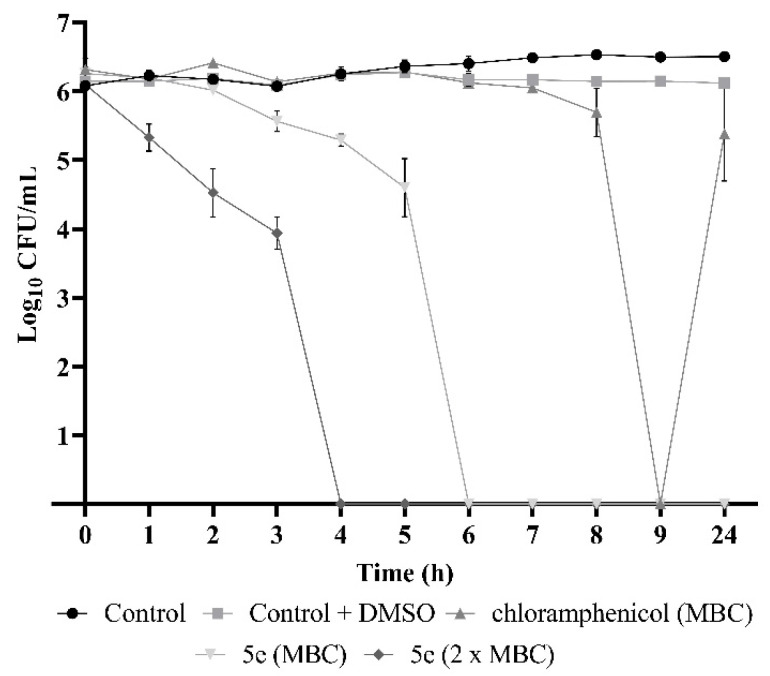
Time–kill curves of MRSA cells exposed to various concentrations of **5e** and chloramphenicol. MBC of **5e** = 3.90 μg/mL; MBC of chloramphenicol = 31.25 μg/mL. MRSA cells incubated in PBS served as growth control, and PBS supplemented with DMSO served as negative control. Values are expressed as means of three independent experiments. Bars indicate SEM.

Time–kill studies revealed that **5e** possesses an important bactericidal potential against the MRSA strain. No viable cells were evidenced starting with 6 h of incubation in the presence of **5e** at a concentration of 3.90 μg/mL (equivalent to an MBC), indicating a total kill effect *(*Figure 5). The same effect was recorded after only 4 h of incubation in PBS supplemented with 7.81 μg/mL (2 × MBC). Moreover, no colonies were observed on MHA after cultivating the samples exposed for 24 h to both tested concentrations, suggesting a significant bactericidal potential of **5e** against MRSA. Chloramphenicol also exhibited bactericidal activity, with a total reduction in CFU count recorded after 9 h of incubation in PBS supplemented with a concentration of 31.25 μg/mL (equivalent to MBC). However, after 24 h, a revival of the MRSA culture was evidenced (Figure 5). No significant differences were recorded between the viability of cells incubated in PBS (growth control) and PBS supplemented with DMSO (negative control).

### 2.5. The Cell Membrane Integrity Was Impaired by ***5e***

**Figure 6:**
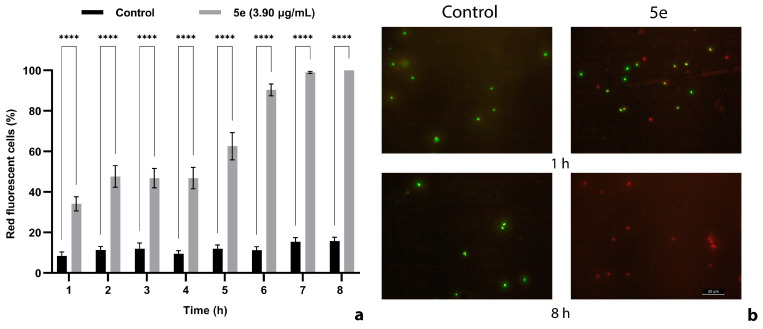
Effect of **5e** exposure on MRSA cell membrane integrity (**a**). Fluorescent images illustrating the impairment of cell membrane disruption effect, visualized by the uptake of the fluorescent nuclear stain, propidium iodide (**b**). Exponential-phase cells were incubated for 8 h in PBS supplemented with **5e** (final concentration equivalent to MBC) and stained with SYTO 9 and propidium iodide. MRSA cells incubated in PBS supplemented with DMSO served as negative control. Red staining indicates the cellular uptake of propidium iodide due to membrane injuries. Green cells are stained with SYTO 9 indicating intact membranes. Values are the mean of three replicates. Bars indicate SEM. Asterisks denote a significant difference (*p* < 0.05) vs. Control (**** = *p* < 0.0001).

The exposure of *S. aureus* medbio1-2012 cells to **5e** resulted in a progressive increase in red-stained cell numbers over time, as fluorescence microscopy revealed (Figure 6). The percentage of control red fluorescent cells was relatively low and constant during the entire incubation time, confirming that propidium iodide (PI) was unable to penetrate non-compromised bacterial membranes. After 6 h of incubation, more than 90% of the exposed cells exhibited red fluorescence, most probably due to the uptake of PI into MRSA cells with damaged membranes (Figure 6a). Higher percentages of red fluorescent cells (99%) were recorded after 7 h of incubation in the presence of **5e** at a concentration of 3.90 µg/mL. At the end of the exposure time (8 h), all MRSA cells were stained in red (Figure 6b).

The biggest challenge in the ongoing fight against MRSA is the ability of this pathogen to rapidly acquire resistance to different antibiotics. Therefore, continuous efforts to develop new effective drugs should be focused on identifying new molecules never used before in therapy, for which MRSA did not come into contact and did not develop yet resistance. A possible solution could be represented by synthetic compounds such as novel tricyclic flavonoids with a methyl group as a substituent on ring A reported here.

Our studies reveal that flavonoids **5a**–**e** exhibited important antibacterial properties against Gram-positive bacteria, especially methicillin-sensitive and -resistant *S. aureus* strains. The lowest MIC (1.95 µg/mL) and MBC (3.90 µg/mL) values were recorded for compound **5e** against an MRSA strain resistant to multiple antibiotics. A milder effect was registered against *Enterococcus faecium* with MIC and MBC values as low as 15.62 µg/mL and 31.25 µg/mL, respectively. Regarding the Gram-negative strains, our flavonoids showed a relatively poor activity, with only one active compound (**5e**) against *E. coli* (MIC = 62.50 µg/mL). The different sensitivity of Gram-positive and Gram-negative bacteria might be explained by the different architecture of the cell wall. Thus, Gram-negative bacteria have an external membrane surrounding the cell wall, containing lipopolysaccharides involved in preventing the penetration of antimicrobials into the cell [18]. However, our results do not confirm the initial hypothesis regarding the enhancement of the activity against Gram-negative bacteria because of the new substitution with a methyl group on the A ring. The MIC values recorded for methyl-substituted flavonoids were higher than or similar to previously reported halogen-substituted tricyclic flavonoids [14,15]. We may presume that the interaction of the positive carbon atom C-2 of the 1,3-dithiol-2-ylium moiety with nucleophilic components of the bacterial membrane, previously presented as the main mechanism of action against both type of bacteria, is more important than increasing the lipophilicity of the A core of flavonoids **5a**–**e**. Furthermore, our previous studies identified a correlation between the nature of substituents on the A ring and the increase in MIC and MBC values in the order of H < F < Cl < Br < I. This was correlated with the size of the substituents and the variation in electronegativity in the halogen series. As the electronegativity decreases, the antimicrobial activities increase, as the outer membrane of bacteria are negatively charged at the physiological pH. This behavior is confirmed by the present study, whereby in tuning substituents at the B ring, the same influence on MIC and MBC values was detected.

The important antibacterial properties of flavonoid **5e** are highlighted by the comparisons with antibiotics used in our experiments as references. Thus, **5e** exhibited a bacteriostatic activity up to 4-fold higher and a bactericidal activity up to 8-fold higher compared with chloramphenicol against MSSA and MRSA strains (except *S. aureus* prxbio2). Also, **5e** exhibited a comparable antimicrobial activity with ampicillin and chloramphenicol against *E. faecium* and *E. coli*. However, the antibacterial activity of most flavonoids was lower compared with the antibiotics used as the control.

Our survey of the literature revealed that **5e** displayed enhanced anti-MRSA activity compared with previously reported natural and synthetic flavonoids. Thus, **5e** exhibited a more significant inhibitory activity against MRSA, with an MIC value up to 420 times smaller compared with rutin, morin, and quercetin [19]. Flavonoid **5e** showed a similar or even higher anti-MRSA effect compared with plant-derived flavones and isoflavones with reported MICs between 1 and 8 μg/mL [20]. Also, **5e** was more active against MRSA strains compared with glabrol, licochalcone A, licochalcone C, and licochalcone E from licorice (MIC_90_ and MIC_50_ between 2 and 8 μg/mL) [21] or tetrahydroxyflavanones isolated from *Sophora exigua* and *Echinosophora koreensis* with MIC values of 3.13 μg/mL and 6.25 μg/mL, respectively [22]. Regarding the bactericidal effect, **5e** showed improved potency compared with diplacone (MBC = 4.9–39.2 µg/mL) [23] or galangin (MBC = 14.16 µg/mL) [24]. A lower bactericidal effect was registered compared with sepicanin A—a new flavanone from *Artocarpus sepicanus* (MBC = 2.9 µg/mL) [25].

Flavonoid **5e** also showed important antifungal activity against *Candida krusei* and *C. albicans*, with MIC and MBC values as low as 31.25 µg/mL. The same compound was more active than fluconazole against both *Candida* strains, with an MBC value recorded for a clinical isolate of *C. krusei* up to 8 times lower compared with the control antifungal. The bactericide and fungicide potential of **5e** suggests a broad antimicrobial spectrum, offering the perspective of important applications. The dual antimicrobial activity of **5e** may open up new possibilities for the development of antimicrobials with both antibacterial and antifungal activities, which are important for addressing the challenges of complex co-infections, reducing the likelihood of resistance developing against multiple pathogens simultaneously.

To confirm the antistaphylococcal potential of **5e**, we selected for further tests a MRSA clinical isolate resistant to cefoxitin, clindamycin, erythromycin, moxifloxacin, penicillin, and tetracycline. The growth kinetic studies revealed an important bacteriostatic effect starting with a concentration as low as 0.97 μg/mL corresponding to ½ MIC which induced a significant 5 h growth delay and a reduced cell growth up to 12 h. Incubating the MRSA cells in MHB supplemented with 1.95 µg/mL (equivalent to an MIC) resulted in a complete inhibition of bacterial growth up to 12 h. No growth was evidenced up to 24 h when MRSA cells were exposed to concentrations of 3.90 µg/mL (2 × MIC), indicating a significant dose-dependent bacteriostatic activity. Moreover, the growth inhibitory effect of the synthetic flavonoid was similar to chloramphenicol, but we need to emphasize that the results are registered at different concentrations. Thus, the **5e** bacteriostatic effect was recorded at a concentration 4 times lower compared with the antibiotic used as reference, suggesting an important antibacterial activity of the tested compound against the MDR MRSA strain.

The time–kill kinetics studies showed that **5e** displayed not only important bacteriostatic activity but also significant bactericidal effects. Thus, all MRSA cells were killed in 6 h of exposure to **5e** at equivalent MBCs (3.90 μg/mL). At higher concentrations (7.81 μg/mL), the complete killing was recorded after only 4 h of exposure. Moreover, no viable cells were evidenced after 24 h of incubation in PBS, suggesting a total kill effect. The bactericidal activity of **5e** was more pronounced compared to chloramphenicol for which a total kill was evidenced after 9 h of exposure at concentrations 8 times higher compared to **5e**. Furthermore, viable MRSA cells exposed to chloramphenicol were evidenced after 24 h of incubation.

The bactericidal potential of **5e** is also confirmed by the fluorescence microscopy results. When a combination of fluorescent dyes (PI and SYTO 9) is used for staining, bacterial cells with normal membranes usually impermeable to PI appear stained in green by SYTO 9. Dead cells with compromised membranes emit red fluorescence due to the PI uptake. Our results show a significant increase over time of red MRSA cells exposed to **5e**. Thus, after 7 h of incubation, 99% of the cells were stained in red and the percentage increased to 100% after 8 h, supporting the important bactericidal activity of **5e**. As presented above (Figure 5), the total kill was recorded for a **5e** concentration equivalent to MBCs after 6 h of incubation, although a similar effect was registered using the fluorescence microscope after 8 h (Figure 6). This discrepancy may be explained by the different densities of the cell suspensions exposed to **5e**: approximately 10^6^ CFUs/mL for the time–kill assay and 10^8^ CFUs/mL for the evaluation of membrane integrity. Fluorescence microscopy also revealed massive membrane injuries favoring the PI uptake and pointing to a membrane-type mechanism of action. Considering our previous results concerning the antibacterial activity of sulfur-containing tricyclic flavonoids with halogen substituents at the benzopyran core [14], we have strong reasons to believe that **5e**’s primary mechanism of action is related to cell membrane impairment. However, we do not exclude the hypothesis of additional action mechanisms being responsible for the antibacterial activity of **5e**; therefore, new investigations are necessary.

Overall, our results support the idea that **5e** possesses important antibacterial properties, highlighting the suitability for the identification of new strategies to mitigate the MRSA burden. However, further studies regarding cytotoxicity are necessary to confirm the potential of **5e** in order to develop new and efficient anti-MRSA agents.

## 3. Materials and Methods

### 3.1. Chemistry

NMR spectra were recorded on a Bruker 500 MHz spectrometer (Bruker BioSpin, Rheinstetten, Germany). Chemical shifts were reported in ppm downfield from TMS. Mass spectra were recorded on a Thermo Scientific ISQ LT instrument (Thermo Fisher Scientific Inc., Waltham, MA, USA). IR spectra were recorded on a Bruker Tensor 27 instrument (Bruker Optik GmbH, Ettlingen, Germany). Melting points were obtained on a KSPI melting point meter (A. KRÜSS Optronic, Hamburg, Germany) and were uncorrected. All reagents were commercially available and used without further purification.

#### 3.1.1. General Procedure for 6-Methyl-2-phenyl-4-oxochroman-3-yl N,N-diethyldithiocarbamate (**4a**)

To a solution of 1-(5-methyl-2-hydroxyphenyl)-1-oxoethan-2-yl *N*,*N*-diethyldithiocarbamate (**2**) (0.15 g, 0.5 mmol) in a mixture of CHCl_3_/MeOH (10 mL, 1:1 *v*/*v*), aminal **3a** (0.13 g, 0.5 mmol) was added and the reaction mixture was heated under reflux for 3 h. After cooling, the solid material was filtered off and purified by recrystallization from ethanol to give **4a** (0.16 g, 81%) as colorless crystals. IR (ATR, cm^−1^) 2745, 1694, 1435, 1251, 1201, 973, 807. ^1^H NMR (CDCl_3_) δ 7.72 (m, 1H), 7.54 (m, 2H), 7.37 (m, 1H), 7.35 (m, 2H), 7.34 (m, 1H), 7.02 (d, *J* = 8.4 Hz, 1H), 5.83 (m, 2H), 3.96 (m, 2H), 3.68 (m, 2H), 2.34 (s, 3H), 1.20 (m, 6H). ^13^C NMR (CDCl_3_) δ 191.9, 188.1, 158.7, 137.7, 136.8, 131.3, 128.7, 128.4, 127.6, 127.2, 120.8, 117.9, 82.7, 59.7, 50.4, 47.2, 20.4, 12.5, 11.4. MS (EI) *m*/*z*: 385.1 (M^+^, 32%) for C_21_H_23_NO_2_S_2_.

#### 3.1.2. 6-Methyl-2-(4-methylphenyl)-4-oxochroman-3-yl N,N-diethyldithiocarbamate (**4b**)

0.15 g, 74%. IR (ATR, cm^−1^) 2960, 1684, 1409, 1249, 1188, 834. ^1^H NMR (CDCl_3_) δ 7.71 (d, *J* = 2.0 Hz, 1H), 7.41 (d, *J* = 8.0 Hz, 2H), 7.35 (dd, *J* = 2.0 Hz, *J* = 8.5 Hz, 1H), 7.16 (d, *J* = 8.0 Hz, 2H), 6.99 (d, *J* = 8.4 Hz, 1H), 5.83 (m, 1H), 5.79 (m, 1H), 3.96 (m, 2H), 3.68 (q, *J* = 7.0 Hz, 2H), 2.34 (s, 3H), 2.33 (s, 3H), 1.21 (m, 6H). ^13^C NMR (CDCl_3_) δ 191.2, 188.2, 158.7, 138.5, 137.6, 133.9, 131.2, 129.1, 127.5, 127.2, 120.7, 117.9, 82.5, 59.7, 50.4, 47.2, 21.2, 20.4, 12.5, 11.4. MS (EI) *m*/*z*: 399.1 (M^+^, 32%) for C_22_H_25_NO_2_S_2_.

#### 3.1.3. 6-Methyl-2-(4-fluorophenyl)-4-oxochroman-3-yl N,N-diethyldithiocarbamate (**4c**)

0.16 g, 78%. IR (ATR, cm^−1^) 3021, 1691, 1431, 1274, 1219, 981. ^1^H NMR (CDCl_3_) δ 7.73 (d, *J* = 2.0 Hz, 1H), 7.52 (d, *J* = 8.5 Hz, 2H), 7.37 (dd, *J* = 2.0 Hz, *J* = 8.4 Hz, 1H), 7.04 (d, *J* = 8.5 Hz, 2H), 6.98 (d, *J* = 8.4 Hz, 1H), 5.88 (m, 1H), 5.76 (m, 1H), 3.95 (m, 2H), 3.68 (m, 2H), 2.34 (s, 3H), 1.21 (m, 6H). ^13^C NMR (CDCl_3_) δ 191.8, 187.9, 163.8, 158.6, 137.7, 132.7, 131.5, 129.6, 127.3, 120.7, 117.8, 115.2, 82.2, 60.1, 50.6, 47.2, 20.4, 12.5, 11.4. MS (EI) *m*/*z*: 403.1 (M^+^, 24%) for C_21_H_22_FNO_2_S_2_.

#### 3.1.4. 6-Methyl-2-(4-chlorophenyl)-4-oxochroman-3-yl N,N-diethyldithiocarbamate (**4d**)

0.17 g, 82%. IR (ATR, cm^−1^) 2967, 1684, 1431, 1298, 878, 547. ^1^H NMR (CDCl_3_) δ 7.72 (d, *J* = 2.0 Hz, 1H), 7.48 (d, *J* = 8.4 Hz, 2H), 7.37 (dd, *J* = 2.0 Hz, *J* = 8.4 Hz, 1H), 7.32 (d, *J* = 8.4 Hz, 2H), 6.99 (d, *J* = 8.4 Hz, 1H), 5.84 (m, 1H), 5.77 (m, 1H), 3.95 (m, 2H), 3.68 (m, 2H), 2.34 (s, 3H), 1.22 (t, *J* = 7.1 Hz, 6H). ^13^C NMR (CDCl_3_) δ 191.7, 187.8, 158.5, 137.7, 135.3, 134.5, 131.5, 129.1, 128.5, 127.2, 120.7, 117.8, 82.1, 59.8, 50.5, 47.2, 20.4, 12.5, 11.4. MS (EI) *m*/*z*: 419.1 (M^+^, 32%) for C_21_H_22_ClNO_2_S_2_.

#### 3.1.5. 6-Methyl-2-(4-bromophenyl)-4-oxochroman-3-yl N,N-diethyldithiocarbamate (**4e**)

0.18 g, 79%. IR (ATR, cm^−1^) 2984, 1684, 1419, 1245, 1201, 808, 508, 437. ^1^H NMR (CDCl_3_) δ 7.72 (d, *J* = 2.0 Hz, 1H), 7.48 (d, *J* = 8.4 Hz, 2H), 7.42 (d, *J* = 8.4 Hz, 2H), 7.37 (dd, *J* = 2.0 Hz, *J* = 8.4 Hz, 1H), 6.99 (d, *J* = 8.4 Hz, 1H), 5.86 (m, 1H), 5.76 (m, 1H), 3.96 (m, 2H), 3.69 (q, *J* = 7.0 Hz, 2H), 2.34 (s, 3H), 1.22 (t, *J* = 7.0 Hz, 6H). ^13^C NMR (CDCl_3_) δ 191.7, 187.7, 158.5, 137.8, 135.8, 131.5, 131.4, 129.4, 127.2, 122.8, 120.7, 117.8, 82.1, 59.7, 50.5, 47.2, 20.4, 12.5, 11.4. MS (EI) *m*/*z*: 463.0 (M^+^, 296%) for C_21_H_22_^79^BrNO_2_S_2_.

#### 3.1.6. General Procedure for 2-N,N-Diethylamino-6-methyl-4-phenyl-4H-1,3-dithiol[4,5-c]chromen-2-ylium Tetrafluoroborate (**5a**)

To a mixture of sulfuric acid (1 mL) and acetic acid (1 mL), flavanone **4a** (0.13 g, 0.33 mmol) was added and the resulting solution was heated to 80 °C for 30 min. The reaction mixture was then left to cool to room temperature, and a solution of sodium tetrafluoroborate (0.3 g) in water (20 mL) was added dropwise with vigorous stirring. The resulting precipitate was filtered, washed with water, and recrystallized from ethanol, yielding the desired tetrafluoroborate **5a** in the form of colorless crystals (0.13 g, 89%). M.p. 258–259 °C. IR (ATR, cm^−1^) 1567, 1441, 1252, 1181, 784. ^1^H NMR (DMSO-*d*6) δ 7.47 (m, 5H), 7.27 (s, 1H), 7.18 (d, *J* = 8.2 Hz, 1H), 6.95 (d, *J* = 8.2 Hz, 1H), 6.71 (s, 1H), 3.93 (m, 2H), 3.84 (m, 2H), 2.28 (s, 3H), 1.39 (t, *J* = 7.1 Hz, 3H), 1.31 (t, *J* = 7.1 Hz, 3H). ^13^C NMR (DMSO-*d*6) δ 184.9, 149.2, 137.4, 132.9, 132.7, 130.3, 129.5, 128.6, 127.9, 127.5, 125.4, 117.5, 116.6, 75.5, 54.7, 54.5, 20.4, 10.7, 10.6. MS (EI) *m*/*z*: 368.1 (M^+^-BF_4_, 8%) for C_21_H_22_NOS_2_]^+^.

#### 3.1.7. 2-N,N-Diethylamino-6-methyl-4-(4-methylphenyl)-4H-1,3-dithiol[4,5-c]chromen-2-ylium Tetrafluoroborate (**5b**)

M.p. 238–240 °C (0.14 g, 88%). IR (ATR, cm^−1^) 1568, 1439, 1247, 1041, 725. ^1^H NMR (DMSO-*d*6) δ 7.38 (d, *J* = 8.1 Hz, 2H), 7.26 (m, 3H), 7.18 (dd, *J* = 1.5 Hz, *J* = 8.3 Hz, 1H), 6.66 (s, 1H), 3.92 (m, 2H), 3.84 (m, 2H), 2.31 (s, 3H), 2.28 (s, 3H), 1.39 (t, *J* = 7.2 Hz, 3H), 1.30 (t, *J* = 7.2 Hz, 3H). ^13^C NMR (DMSO-*d*6) δ 185, 149.3, 139.9, 134.5, 132.9, 132.7, 130.5, 128.7, 128.0, 127.9, 125.3, 117.5, 116.6, 75.5, 54.6, 54.5, 21.2, 20.4, 10.8, 10.6. MS (EI) *m*/*z*: 382.1 (M^+^-BF_4_,9%) for C_22_H_24_NOS_2_]^+^.

#### 3.1.8. 2-N,N-Diethylamino-6-methyl-4-(4-fluorophenyl)-4H-1,3-dithiol[4,5-c]chromen-2-ylium Tetrafluoroborate (**5c**)

M.p. 228–229 °C (0.13 g, 84%). IR (ATR, cm^−1^) 1561, 1430, 1220, 1041, 681 ^1^H NMR (DMSO-*d*6) δ 7.55 (m, 2H), 7.29 (m, 3H), 7.18 (d, *J* = 8.3 Hz, 1H), 6.95 (d, *J* = 8.3 Hz, 1H), 6.74 (s, 1H), 3.93 (m, 2H), 3.84 (m, 2H), 2.28 (s, 3H), 1.40 (t, *J* = 7.2 Hz, 3H), 1.32 (t, *J* = 7.2 Hz, 3H). ^13^C NMR (DMSO-*d*6) δ 184.9, 164.1, 149.0, 133.7, 133.0, 132.8, 130.4, 128.7, 127.4, 125.4, 117.5, 116.6, 116.4, 74.7, 54.6, 54.5, 20.4, 10.8, 10.6. MS (EI) *m*/*z*: 386.1 (M^+^-BF_4_, 5%) for C_21_H_21_FNOS_2_]^+^.

#### 3.1.9. 2-N,N-Diethylamino-6-methyl-4-(4-chlorophenyl)-4H-1,3-dithiol[4,5-c]chromen-2-ylium Tetrafluoroborate (**5d**)

M.p. 192–194 °C (0.14 g, 85%). IR (ATR, cm^−1^) 1540, 1451, 1242, 1051, 744, 567. ^1^H NMR (DMSO-*d*6) δ 7.52 (m, 4H), 7.28 (d, *J* = 1.3 Hz, 1H), 7.19 (dd, *J* = 1.3 Hz, *J* = 8.3 Hz, 1H), 6.96 (d, *J* = 8.3 Hz, 1H), 6.75 (s, 1H), 3.93 (m, 2H), 3.85 (m, 2H), 2.28 (s, 3H), 1.40 (t, *J* = 7.2 Hz, 3H), 1.32 (t, *J* = 7.2 Hz, 3H). ^13^C NMR (DMSO-*d*6) δ 184.9, 149.0, 136.3, 134.9, 133.0, 132.9, 129.9, 129.5, 128.8, 127.0, 125.4, 117.5, 116.5, 74.7, 54.7, 54.5, 20.4, 10.8, 10.6. MS (EI) *m*/*z*: 402.1 (M^+^-BF_4_, 8%) for C_21_H_21_ClNOS_2_]^+^.

#### 3.1.10. 2-N,N-Diethylamino-6-methyl-4-(4-bromophenyl)-4H-1,3-dithiol[4,5-c]chromen-2-ylium Tetrafluoroborate (**5e**)

M.p. 198–200 °C (0.15 g, 83%). IR (ATR, cm^−1^) 1551, 1443, 1254, 1081, 894, 611, 545. ^1^H NMR (DMSO-*d*6) δ 7.66 (d, *J* = 8.4 Hz, 2H), 7.45 (d, *J* = 8.4 Hz, 2H), 7.28 (d, *J* = 1.3 Hz, 1H), 7.18 (dd, *J* = 1.3 Hz, *J* = 8.3 Hz, 1H), 6.96 (d, *J* = 8.3 Hz, 1H), 6.74 (s, 1H), 3.93 (m, 2H), 3.86 (m, 2H), 2.28 (s, 3H), 1.40 (t, *J* = 7.2 Hz, 3H), 1.32 (t, *J* = 7.2 Hz, 3H). ^13^C NMR (DMSO-*d*6) δ 184.9, 149.0, 136.7, 133.1, 132.9, 132.5, 130.1, 128.8, 127.0, 125.4, 123.6, 117.5, 116.5, 74.7, 54.7, 54.5, 20.4, 10.8, 10.6. MS (EI) *m*/*z*: 446.0 (M^+^-BF_4_, 9%) for C_21_H_21_^79^BrNOS_2_]^+^.

### 3.2. Microbial Strains and Culture Media

The synthesized compounds were tested for antimicrobial activity against the following strains: *Staphylococcus aureus* ATCC 25923 (methicillin-sensitive—MSSA), *Escherichia coli* ATCC 25922, *Pseudomonas aeruginosa* PAO1, and *Candida albicans* ATCC 10231 (purchased from local distributors); *Acinetobacter pittii* Cl2 (resistant to ampicillin and chloramphenicol) was isolated from a waste water sample; *S. aureus* prxbio2 (resistant to methicillin—MRSA, cefoxitin, clindamycin, erythromycin, and tetracycline), *S. aureus* medbio1-2012 (resistant to methicillin—MRSA, cefoxitin, clindamycin, erythromycin, moxifloxacin, penicillin, and tetracycline), *Enterococcus faecium* medbio2-2012 (resistant to methicillin, ampicillin, ciprofloxacin, gentamicin, levofloxacin, nitrofurantoin, and penicillin), and *Candida krusei* Prx strains (resistant to fluconazole) were provided by med. biol. PhD Simona Matiut from Praxis Clinical Laboratory, Iasi, Romania.

Each strain was stored at −80 °C in 15% glycerol stocks. Mueller–Hinton agar (MHA, Accumix, Geel, Belgium) and Sabouraud dextrose agar (SDA, Roth, Germany) were used for cultivation of bacterial and fungal strains after removal from the freezer. All strains were cultivated overnight at 37 °C in aerobic conditions. The source of inoculum for each experiment was represented by one colony considered typical for each strain and inoculated in 10 mL of Mueller–Hinton broth (MHB, Roth, Germany) or 10 mL of Sabouraud dextrose broth (SDB, Roth, Germany). All precultures were incubated for 24 h at 37 °C and 190 rpm (for bacterial strains) or 130 rpm (for fungal strains).

### 3.3. Minimum Inhibitory and Bactericidal/Fungicidal Concentration Determination

A microdilution method was employed to determine the minimum inhibitory concentration (MIC) and minimum bactericidal/fungicidal concentration (MBC/MFC), following a previously presented protocol [14,26]. Bacterial and fungal suspensions were adjusted to approximately 2 × 10^6^ CFUs/mL (CFUs = colony-forming units) and 1 × 10^3^ CFUs/mL, respectively, and used to inoculate each well of a microplate containing the corresponding culture medium. Each tested flavonoid was diluted using DMSO (Merck, Darmstadt, Germany) to obtain a concentration range of 0.12 to 250 µg/mL. Negative control was represented by DMSO (concentrations ranging from 0.012 to 24.87% *v*/*v*). Inoculated MHB or SDB medium served as growth control. Ampicillin, chloramphenicol, ciprofloxacin, kanamycin, and fluconazole were used as reference antimicrobials. The lowest concentration of the tested flavonoids showing no visible microbial growth after a 24 h incubation was considered as the MIC. MBC and MFC were considered as the lowest concentrations with no colony growth after plating samples taken from MIC assay wells onto MHA or SDA.

### 3.4. Growth Inhibition Assay

The growth of *S. aureus* medbio1-2012 strain was assessed in the presence of **5e** for 24 h using a previous described method, with minor modifications [14,26]. Inoculum (final cell density of approximately 10^6^ CFUs/mL) was added in 15 mL MHB supplemented with **5e** at final concentrations equivalent to ½ MIC, MIC, and 2 × MIC. Positive control was represented by inoculated MHB supplemented with chloramphenicol (½ MIC, MIC, and 2 × MIC). DMSO added to the inoculated culture medium (final concentrations equivalent to those used to prepare the samples) served as negative control. The growth control was represented by inoculated MHB alone. Samples were taken during incubation (37 °C and 190 rpm) every hour until 12 h and then at 24 h. A bacterial growth curve was constructed by plotting the mean optical densities (OD at 600 nm) over time to assess the bacterial growth.

### 3.5. Time–Kill Assay

The bactericidal potential of **5e** against *S. aureus* medbio1-2012 was evaluated using a time–kill kinetic study, as described earlier [14]. Briefly, a bacterial suspension in PBS (final cell density of approximately 10^6^ CFUs/mL) was supplemented with **5e** (final concentration equivalent to MBC and 2 × MBC). Chloramphenicol added in PBS at a concentration equivalent to MBC served as positive control, and PBS supplemented with DMSO (final concentrations equivalent to those used to prepare **5e** corresponding MBC and 2 × MBC) served as negative control. Samples were taken every hour up to 9 h, and at 24 h, were plated onto MHA after performing a tenfold serial dilution. Following an incubation at 37 °C for 24 h, the number of CFUs was determined and the final values (CFUs/mL) were transformed into log_10_ values. The bactericidal effect was considered as a ≥3 log_10_ reduction in the total CFUs/mL from the initial inoculum. Mean CFU counts versus time were plotted to construct a viability curve.

### 3.6. Assessment of Membrane Integrity by Propidium Iodide Uptake

The membrane integrity of the *S. aureus* medbio1-2012 cells was evaluated using the Live/Dead BacLight bacterial viability kit (Invitrogen, Waltham, MA, USA) and fluorescence microscopy, following a previously described procedure [14]. Briefly, an exponential phase cell suspension (approximately 10^8^ CFUs/mL) was prepared in PBS supplemented with **5e** (final concentration 3.90 µg/mL, equivalent to MBC) and incubated at 37 °C and 190 rpm for 8 h. Cells in PBS supplemented with DMSO at the same concentration used to prepare the sample served as negative control. Samples taken every hour were stained with SYTO 9 and propidium iodide (PI), according to the manufacturers’ specifications. The cell count was performed using a DM100 LED fluorescence microscope (Leica, Wetzlar, Germany) and an I3 blue excitation range filter cube (BP 450 ± 490 nm band-pass filter). For each sample, 10 random images were captured which were used further to calculate the ratio between green/red fluorescent cells and total cells as percentage.

### 3.7. Statistical Analysis

Each experiment was repeated at least three times. Dunnett’s multiple comparisons test was used for the evaluation of the results. All data were analyzed using GraphPad Prim 9 software (GraphPad Software, Inc., La Jolla, CA, USA) and presented as mean (n = 3) ± SEM. Differences between groups were considered significant when *p* < 0.05.

## 4. Conclusions

Novel tricyclic flavonoids were synthesized and tested for antimicrobial activities against selected bacterial and fungal strains. The structures of novel flavonoids and of their 3-dithiocarbamic flavanones were unambiguously proven by analytical and spectral data. X-ray structural analyses of two compounds confirm the structures of the investigated flavonoids. Our results show that the tricyclic flavonoids exhibited important antimicrobial activity, especially against Gram-positive bacteria, with flavonoid **5e** as the most active compound. Flavonoid **5e** displayed significantly higher bacteriostatic and bactericidal effects compared to chloramphenicol against an MDR MRSA strain. This compound induced a total kill effect up to 24 h at very low concentrations most probably due to the impairment of the cell membrane. All the data support the idea that flavonoid **5e** could be a reliable candidate to develop effective anti-MRSA agents, but further studies regarding the mechanism of action and cytotoxicity are necessary.

## Figures and Tables

**Table 1:** Coupling constants and diastereoisomer ratio of flavonones **4**.

Flavanones 4	a	b	c	d	e
^3^*J*_H2–H3_ *syn* (Hz)	3.4	3.2	3.9	3.7	3.7
^3^*J*_H2–H3_ *anti* (Hz)	7.8	8.1	8.7	8.3	8.3
*syn: anti* ratio	31:69	12:88	11:89	12:88	10:90

**Table 2:** Minimum inhibitory concentrations of **5a**–**e** flavonoids against tested microbial strains (µg/mL).

Microbial Strains	5a	5b	5c	5d	5e	Control	DMSO (%)
*Staphylococcus aureus* ATCC 25923	7.81	7.81	7.81	3.90	3.90	1.95 ^a^/7.81 ^chl^	24.87
*S. aureus* prxbio2	7.81	15.62	15.62	15.62	15.62	7.81 ^chl^	24.87
*S. aureus* medbio1-2012	15.62	3.90	15.62	3.90	1.95	7.81 ^chl^	24.87
*Enterococcus faecium* medbio2-2012	62.50	31.25	62.50	31.25	15.62	15.62 ^chl^	12.43
*Escherichia coli* ATCC 25922	125	125	125	125	62.50	62.50 ^a^/7.81 ^k^	12.43
*Acinetobacter pittii* Cl2	62.50	62.50	62.50	62.50	62.50	>250 ^a^/0.37 ^cip^	6.21
*Pseudomonas aeruginosa* PAO1	125	125	125	125	125	>250 ^a^	12.43
*Candida krusei* Prx	62.50	31.25	62.50	31.25	31.25	62.50 ^f^	6.21
*C. albicans* ATCC 10231	62.50	31.25	62.50	31.25	31.25	>500 ^f^	6.21

^a^—ampicillin; ^chl^—chloramphenicol; ^cip^—ciprofloxacin; ^k^—kanamycin; ^f^—fluconazole. The values are means of at least three replicates.

**Table 3:** Minimum bactericidal/fungicidal concentrations of **5a–e** flavonoids against tested microbial strains (µg/mL).

Microbial Strains	5a	5b	5c	5d	5e	Control
*Staphylococcus aureus* ATCC 25923	31.25	15.62	31.25	31.25	31.25	7.81 ^a^
*S. aureus* prxbio2	250	62.50	250	250	62.50	125 ^chl^
*S. aureus* medbio1-2012	62.50	7.81	31.25	15.62	3.90	31.25 ^chl^
*Enterococcus faecium* medbio2-2012	125	62.50	62.50	62.50	31.25	>250 ^chl^
*Escherichia coli* ATCC 25922	125	125	125	125	125	125 ^a^
*Acinetobacter pittii* Cl2	125	125	125	125	125	>250 ^a^
*Pseudomonas aeruginosa* PAO1	>250	>250	>250	>250	>250	>250 ^a^
*Candida krusei* Prx	125	31.25	125	62.50	31.25	62.50 ^f^
*C. albicans* ATCC 10231	125	62.50	125	62.50	62.50	>500 ^f^

^a^—ampicillin; ^chl^—chloramphenicol; ^f^—fluconazole. The values are means for at least three replicates.

## Data Availability

Data are contained within the article and supplementary materials.

## Supplementary Materials

The following supporting information can be downloaded at https://www.mdpi.com/article/10.3390/ph17101276/s1. Elemental analysis data for compounds **4a**–**e** and **5a**–**e**. Copies of 13C-NMR spectra. Details of X-ray structure determinations. Additionally, complete crystallographic data have been deposited with the Cambridge Crystallographic Data Centre under the numbers CCDC 2375914 (**4a**) and 2375915 (**5c**). Copies of the data can be obtained free of charge from www.ccdc.cam.ac.uk/data_request/cif, accessed on 25 September 2024.
