# Supplementary material for: Novel Tricyclic Flavonoids as Promising Anti-MRSA Agents
Source: Pharmaceuticals (Basel). 2024 Sep 26;17(10):1276. doi: 10.3390/ph17101276 (PMC11510641; doi:10.3390/ph17101276)
Supplement: Supplementary file 1 [file pharmaceuticals-17-01276-s001.zip › pharmaceuticals-3214264-supplementary.pdf]

Novel tricyclic flavonoids as promising anti-MRSA agents

Cristina-Veronica Moldovan, Loredana-Elena Mantea, Mihaela Savu, Peter G. Jones, Laura Gabriela Sarbu, Marius Stefan and Mihail Lucian Birsa

Supplementary Material

1. Crystal structure determinations **S2**

2. Elemental analysis **S4**

3. Copies of ^{13}C NMR spectra **S5**

Crystal Structure Determinations

Crystals were mounted in inert oil on Hampton loops and transferred to the cold gas stream of a Rigaku/OD XtaLAB Synergy diffractometer. Mirror-focussed Mo-$K\alpha$ radiation was employed for the intensity measurements. Absorption corrections were implemented on the basis of multi-scans. The structures were refined anisotropically on F^2 using the program SHELXL-2019. Hydrogen atoms were included using rigid methyl groups or a riding model starting from calculated positions.

*Exceptions/special details for compound **4a***: The two independent molecules are related by a pseudo inversion centre at (0.25, 0.25, 0.25), corresponding after appropriate transformation to the pseudo *I*-centring. The "extra" reflections corresponding to the larger cell are weak, but definitely present. In both independent molecules, the NEt_2 group is disordered over two positions; the minor components have low occupation factors [0.049(2) and 0.1236(2) respectively]. Appropriate restraints were employed to improve refinement stability, but the dimensions of disordered groups (and especially the minor components) should be interpreted with caution. Six badly fitting reflections (errors > 8σ) were omitted from the refinement.

Crystallographic data are summarized in Table S1. Additionally, complete data have been deposited with the Cambridge Crystallographic Data Centre under the numbers CCDC 2375914 & 2375915. Copies of the data can be obtained free of charge from www.ccdc.cam.ac.uk/data_request/cif. Ellipsoid plots are given in the main paper.

Table S1: Crystallographic data and structure refinement details.

Compound	**4a**	**5c**
CCDC number	2375914	2375915
Formula	$C_{21}H_{23}NO_2S_2$	$C_{21}H_{21}BF_5NOS_2$
M_r	385.52	473.32
Cryst. size (mm)	0.2 x 0.15 x 0.1	0.15 x 0.15 x 0.1
Crystal system	triclinic	triclinic
Space group	*P*(-1)	*P*(-1)
Temperature (°C)	-173	-173
a (Å)	10.59059(19)	10.0620(2)
b (Å)	13.2084(3)	10.3095(2)
c (Å)	15.2224(3)	10.5119(3)
α (°)	70.4771(18)	83.5594(18)
β (°)	83.7568(16)	81.470(2)
γ (°)	75.5757(16)	75.0396(18)
V (Å^3)	1943.32	1038.72
Z	4	2
D_x (Mg m^{-3})	1.318	1.513
λ (Å)	0.71073	0.71073
μ (mm^{-1})	0.29	0.32
Transmissions	0.903 – 1.000	0.914 – 1.000
F(000)	816	488
$2\theta_{max}$	82.6	82.8
Refl. measured	248986	132855
Refl. indep.	25453	13633
R_{int}	0.034	0.031
Parameters	517	283
Restraints	44	0
$wR(F^2$, all refl.)	0.117	0.105
$R(F, >4\sigma(F))$	0.038	0.035
S	1.05	1.02
Max. Δ*p* (e Å^{-3})	1.33, –0.44	1.24, –0.52

Elemental analysis

Elemental analyses (C, H) were conducted using a CE440 Elemental Analyser; the results were found to be in good agreement (±0.3%) with the calculated values.

Table S2. Elemental analysis data for compounds **4** and **5**.

Compound	% C		% H	
	**calcd.**	**found**	**calcd.**	**found**
**4a**	65.42	65.63	6.01	5.88
**4b**	66.13	66.29	6.31	6.02
**4c**	62.50	62.78	5.50	5.40
**4d**	60.06	60.24	5.28	5.01
**4e**	54.31	54.53	4.77	4.51
**5a**	55.39	55.64	4.87	4.73
**5b**	60.41	60.68	5.54	5.38
**5c**	53.29	53.57	4.47	4.31
**5d**	51.50	51.71	4.32	4.08
**5e**	47.21	47.43	3.96	3.84

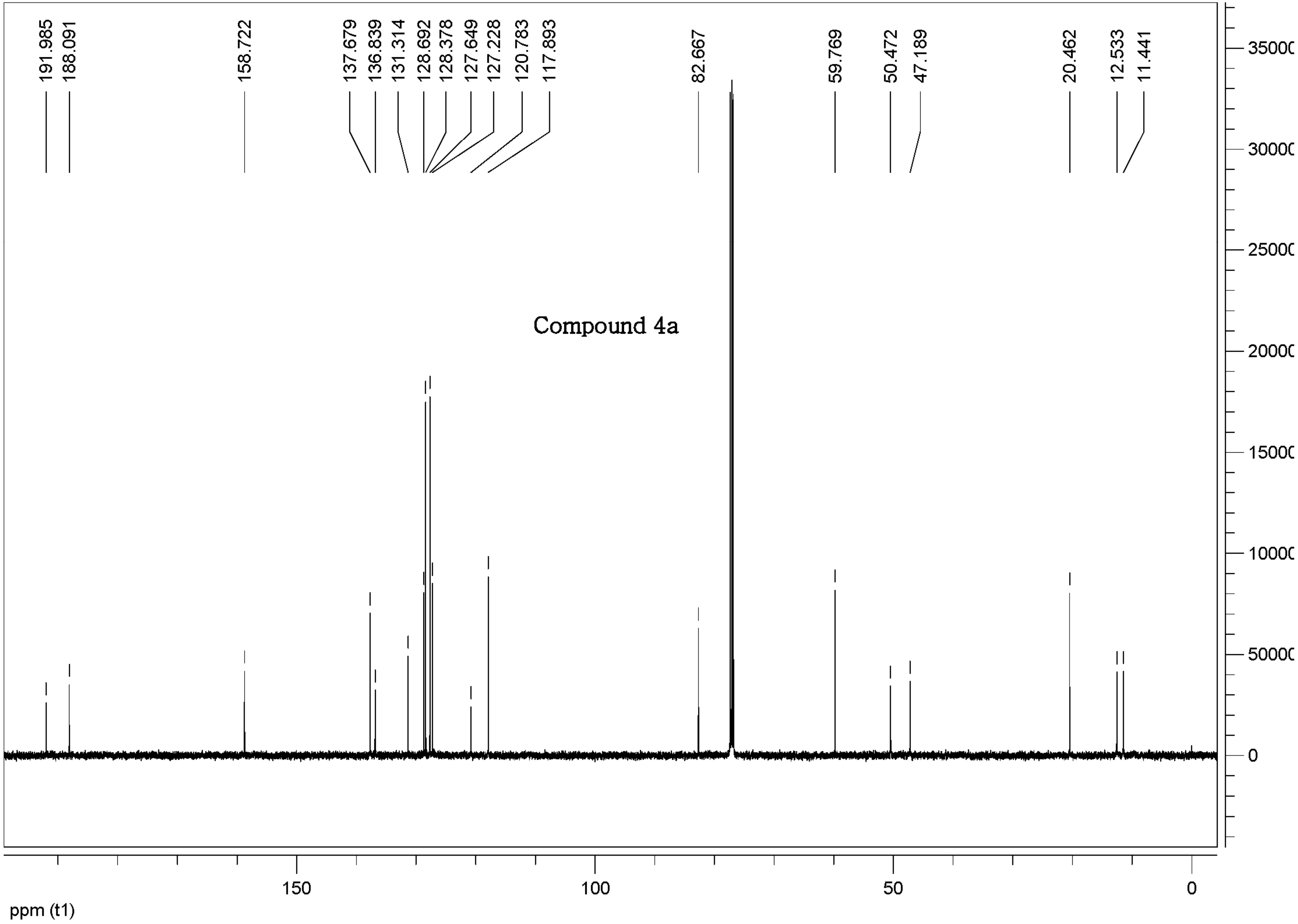
191.985
188.091
158.722
137.679
136.839
131.314
128.692
128.378
127.649
127.228
120.783
117.893
82.667
59.769
50.472
47.189
20.462
12.533
11.441
Compound 4a
ppm (t1)
150
100
50
0

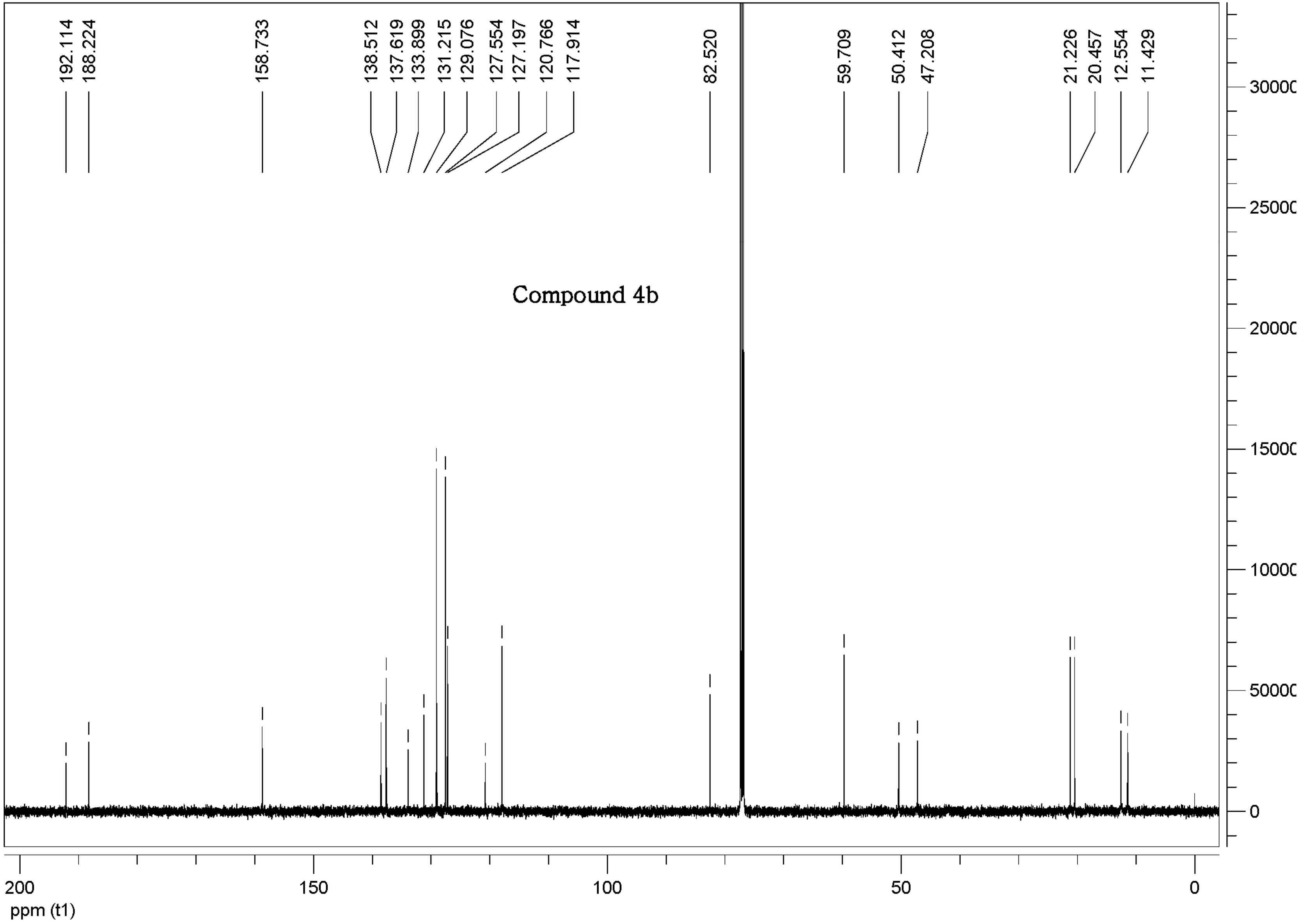
192.114
188.224
158.733
138.512
137.619
133.899
131.215
129.076
127.554
127.197
120.766
117.914
82.520
59.709
50.412
47.208
21.226
20.457
12.554
11.429
Compound 4b
200
150
100
50
0
ppm (t1)

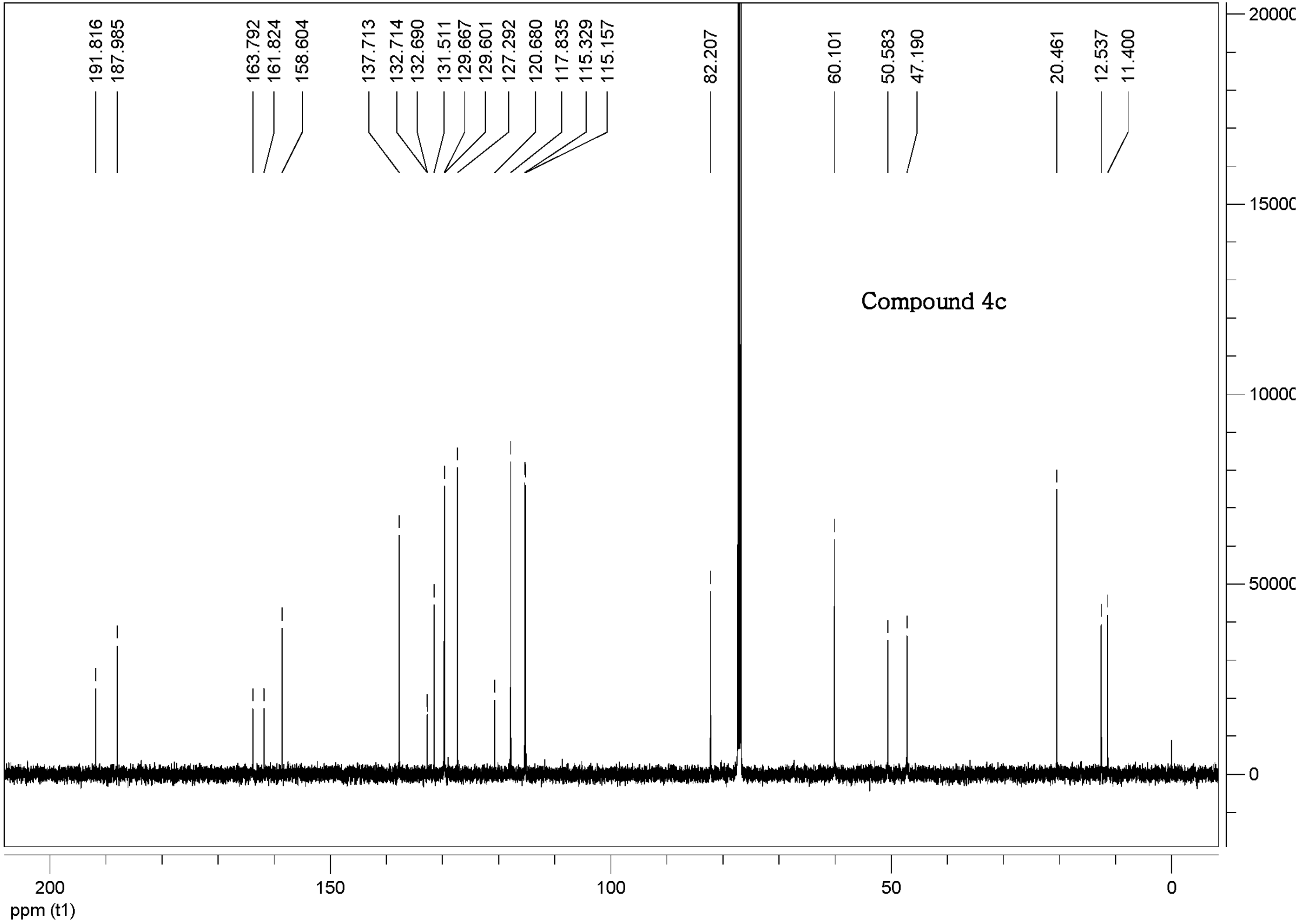

191.816
187.985
163.792
161.824
158.604
137.713
132.714
132.690
131.511
129.667
129.601
127.292
120.680
117.835
115.329
115.157
82.207
60.101
50.583
47.190
20.461
12.537
11.400
Compound 4c
200
150
100
50
0
ppm (t1)

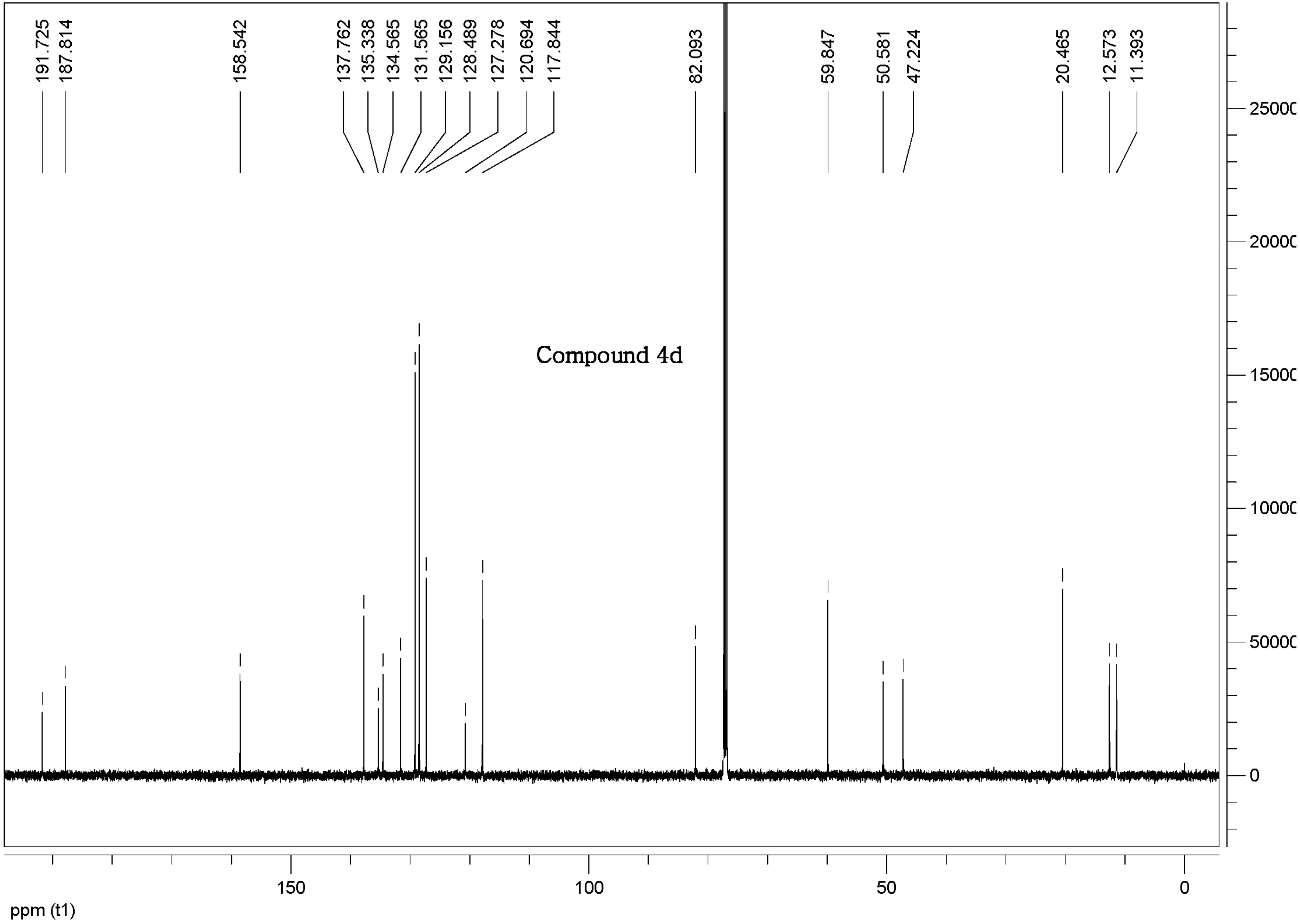
191.725
187.814
158.542
137.762
135.338
134.565
131.565
129.156
128.489
127.278
120.694
117.844
82.093
59.847
50.581
47.224
20.465
12.573
11.393
Compound 4d
25000
20000
15000
10000
50000
0
150
100
50
0
ppm (t1)

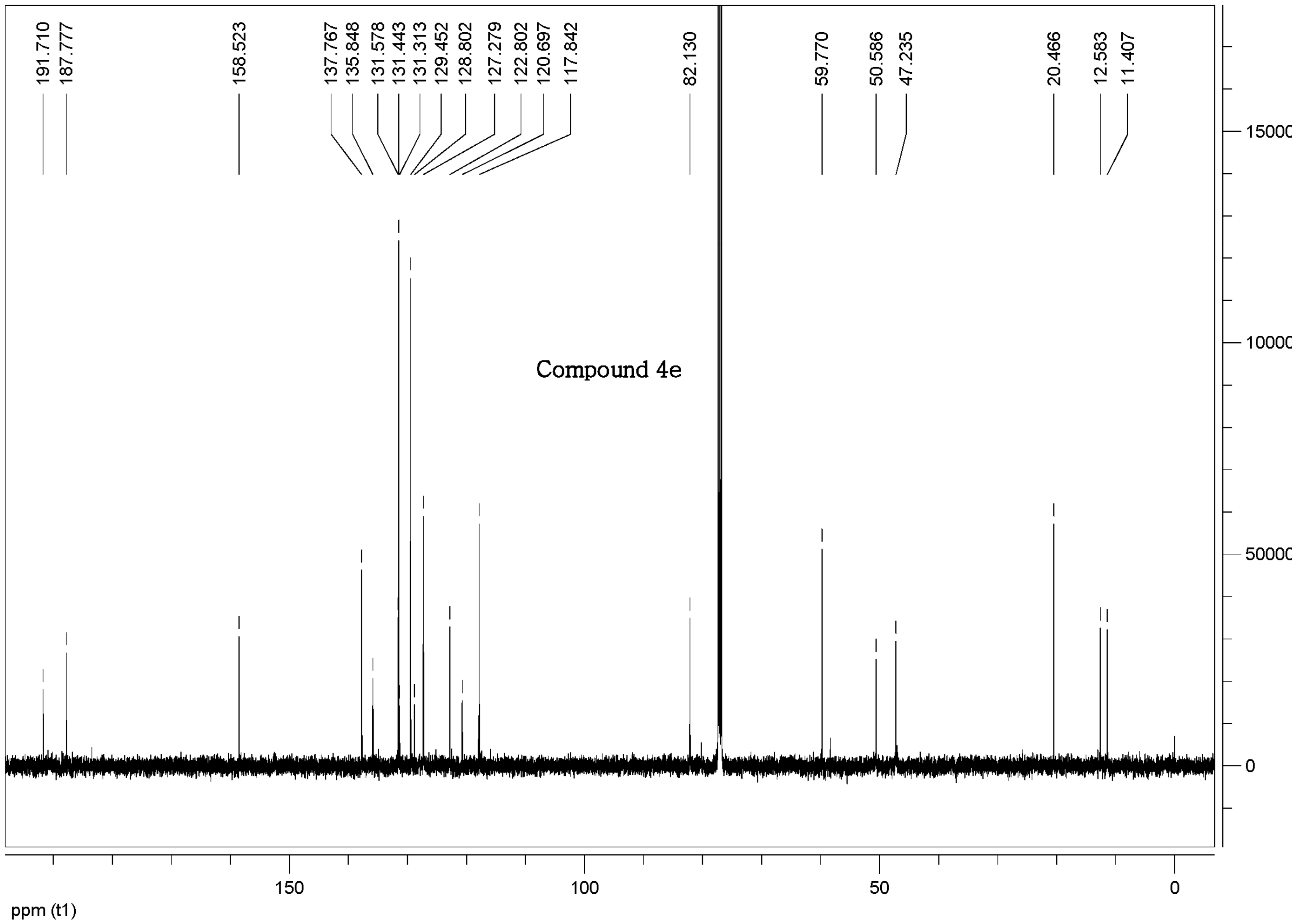
191.710
187.777
158.523
137.767
135.848
131.578
131.443
131.313
129.452
128.802
127.279
122.802
120.697
117.842
82.130
59.770
50.586
47.235
20.466
12.583
11.407
Compound 4e
15000
10000
50000
0
150
100
50
0
ppm (t1)

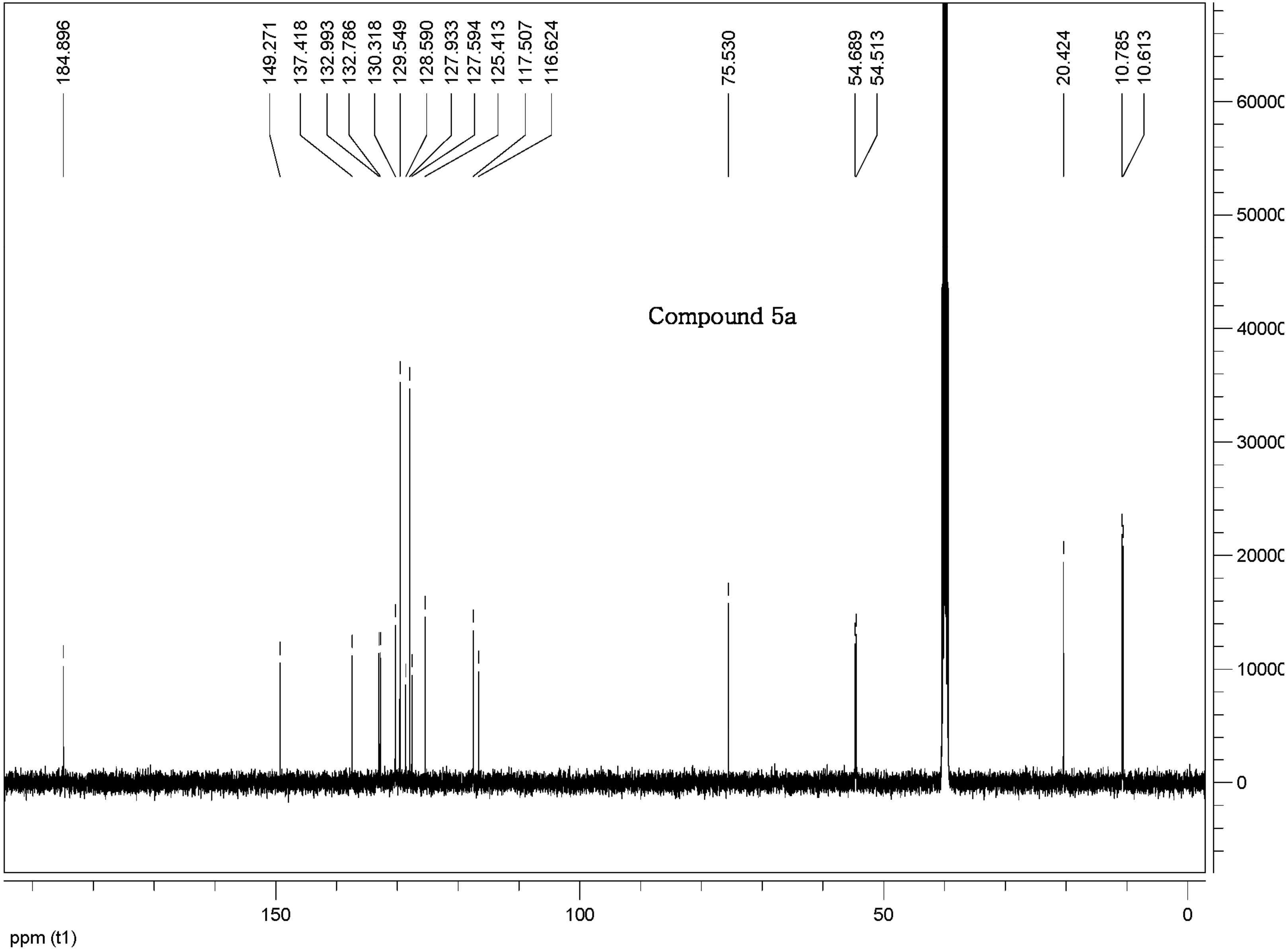
184.896
149.271
137.418
132.993
132.786
130.318
129.549
128.590
127.933
127.594
125.413
117.507
116.624
75.530
54.689
54.513
20.424
10.785
10.613
Compound 5a
60000
50000
40000
30000
20000
10000
0
150
100
50
0
ppm (t1)

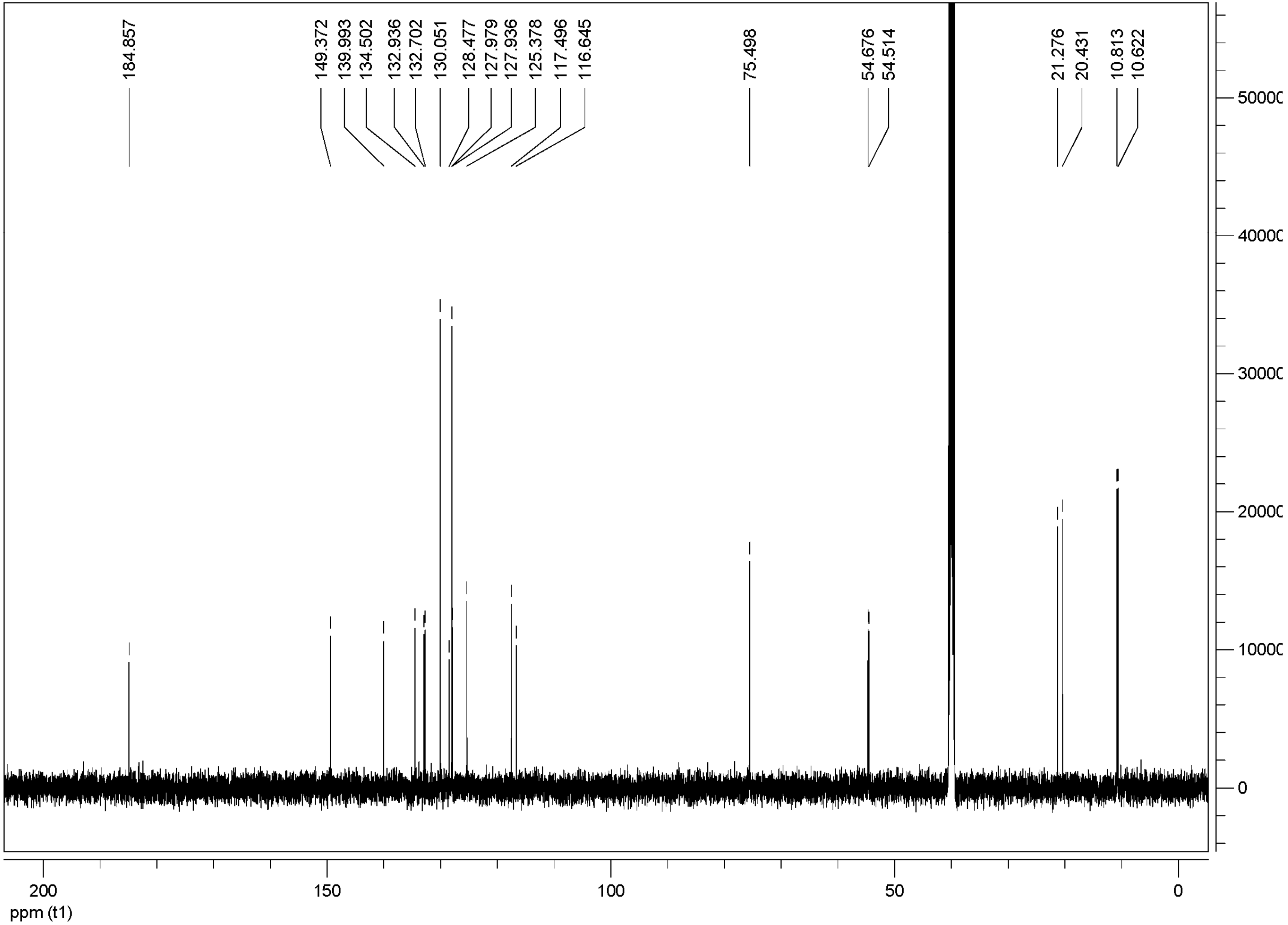
184.857
149.372
139.993
134.502
132.936
132.702
130.051
128.477
127.979
127.936
125.378
117.496
116.645
75.498
54.676
54.514
21.276
20.431
10.813
10.622
50000
40000
30000
20000
10000
0
200
150
100
50
0
ppm (t1)

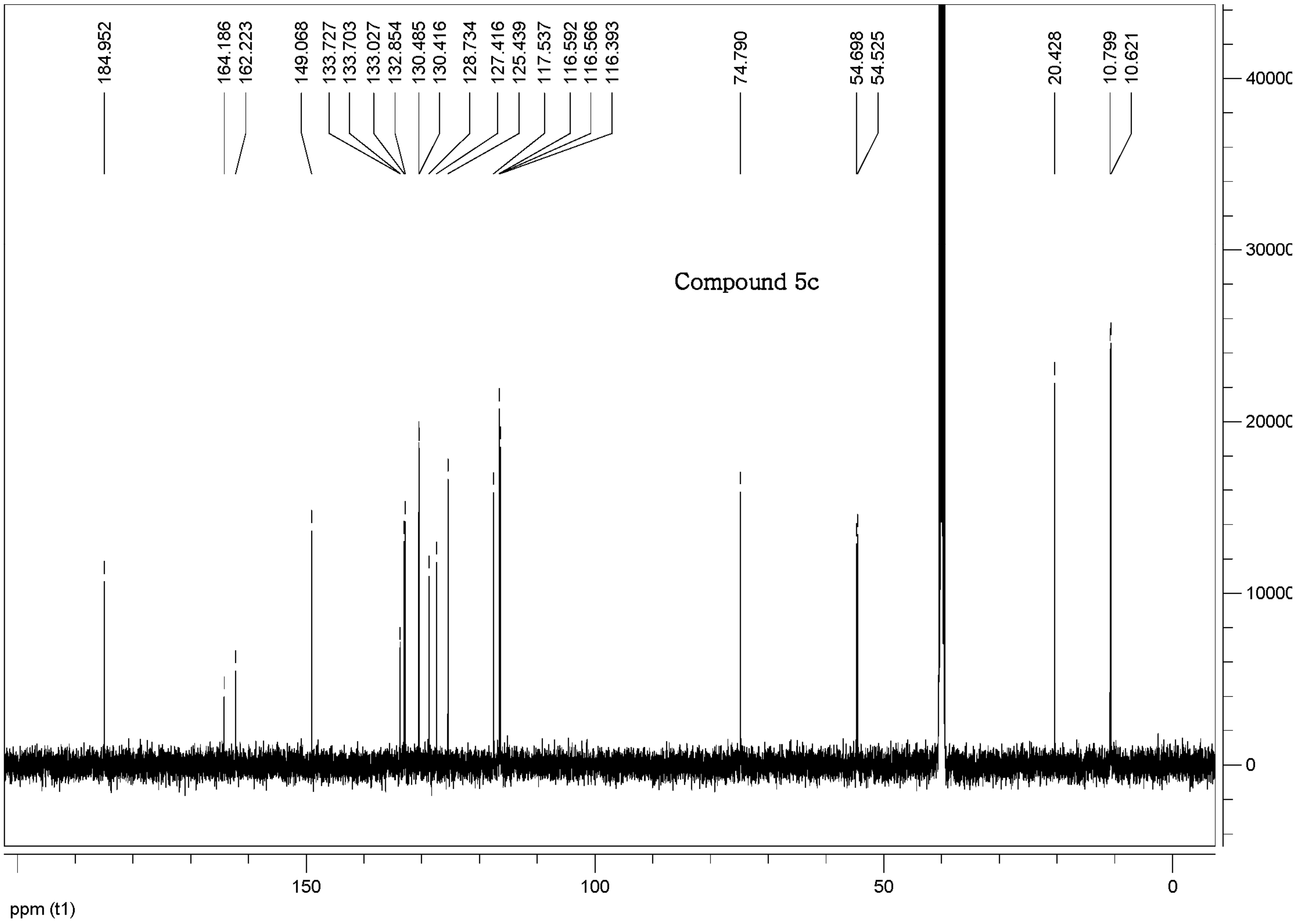
184.952
164.186
162.223
149.068
133.727
133.703
133.027
132.854
130.485
130.416
128.734
127.416
125.439
117.537
116.592
116.566
116.393
74.790
54.698
54.525
20.428
10.799
10.621
Compound 5c
ppm (t1)
150
100
50
0

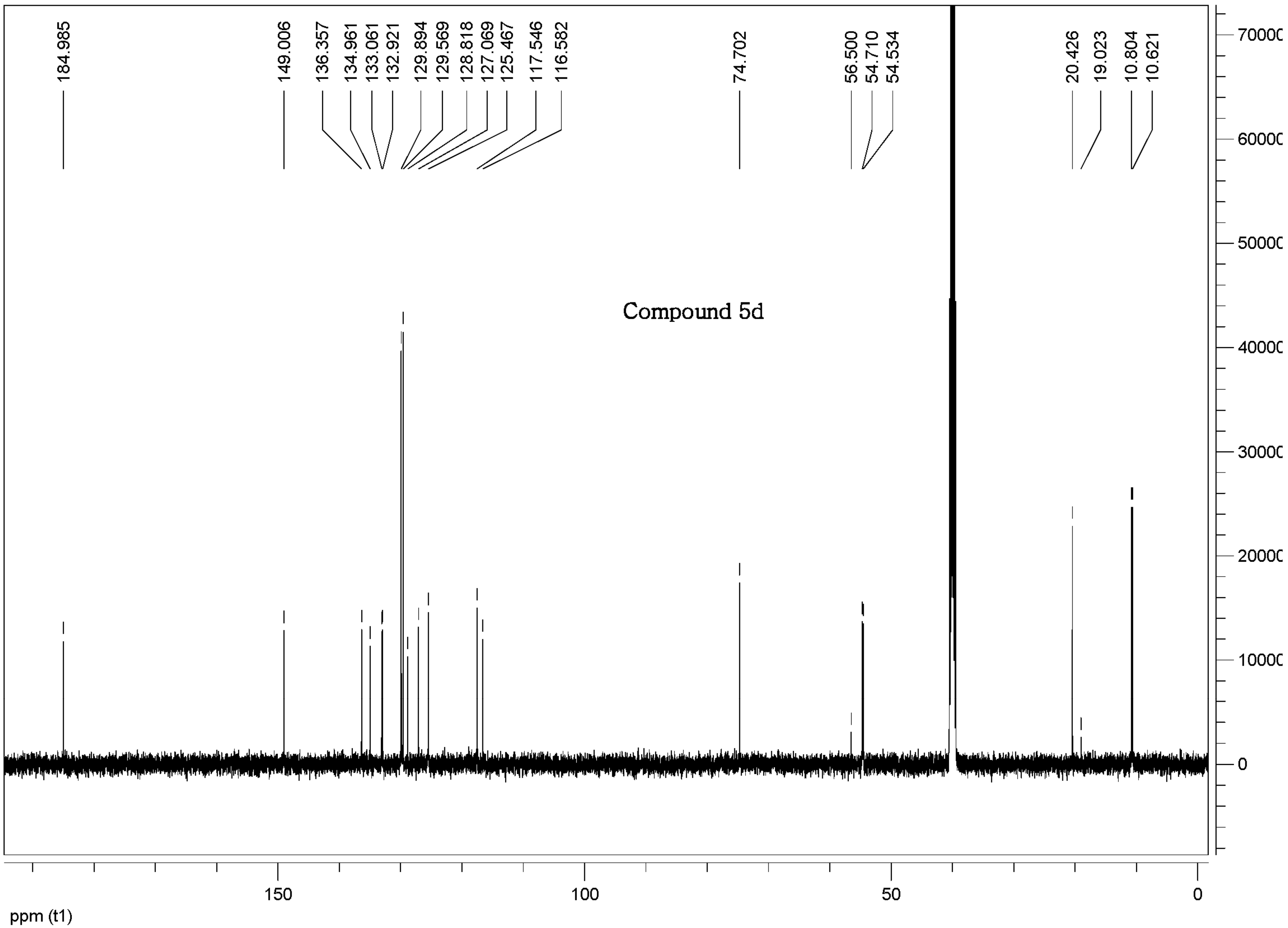
184.985
149.006
136.357
134.961
133.061
132.921
129.894
129.569
128.818
127.069
125.467
117.546
116.582
74.702
56.500
54.710
54.534
20.426
19.023
10.804
10.621
Compound 5d
150
100
50
0
ppm (t1)

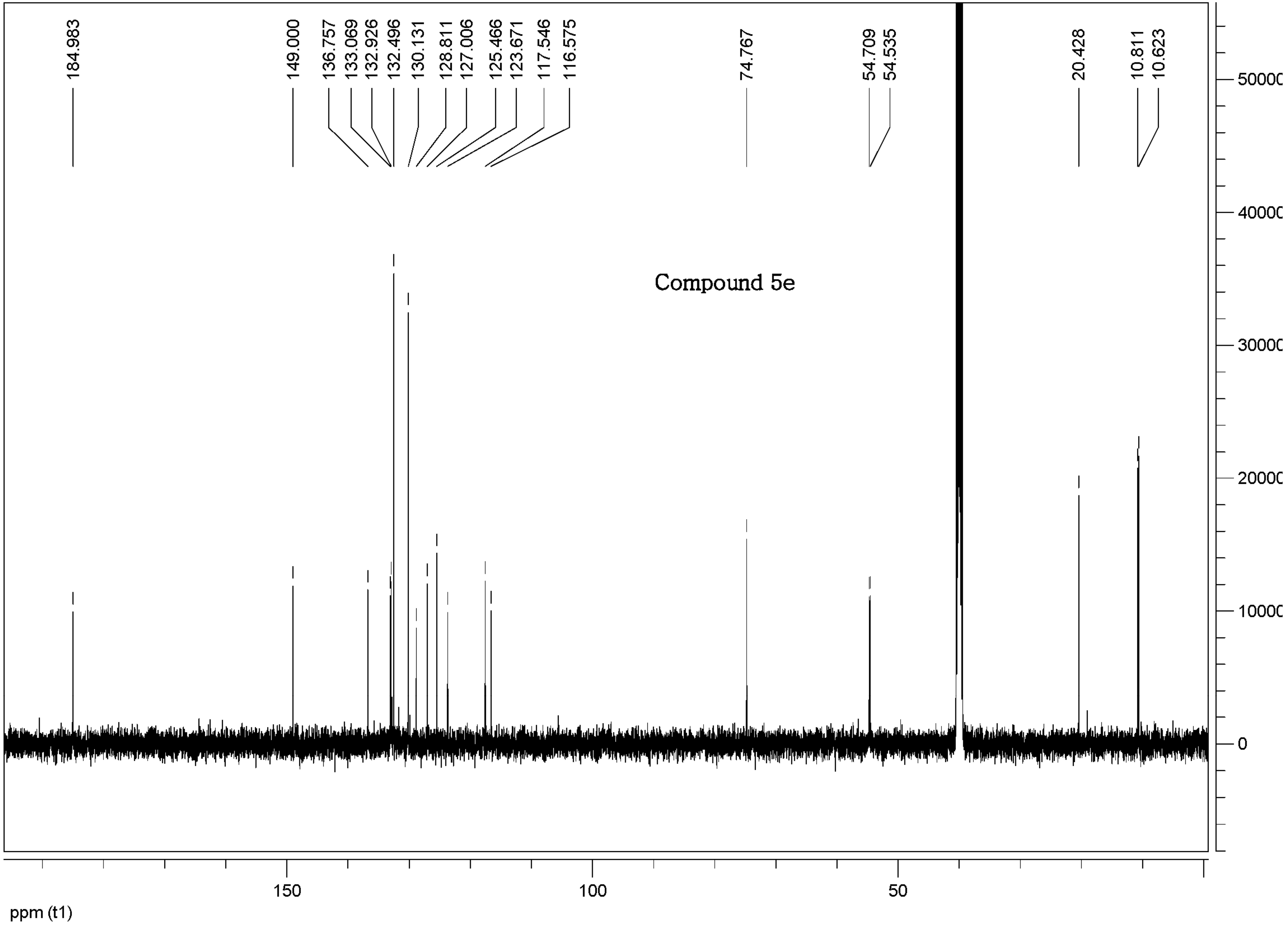

184.983
149.000
136.757
133.069
132.926
132.496
130.131
128.811
127.006
125.466
123.671
117.546
116.575
74.767
54.709
54.535
20.428
10.811
10.623
Compound 5e
50000
40000
30000
20000
10000
0
150
100
50
ppm (t1)
